# Case 5/2018 - Severe Pulmonary Valve Stenosis (PVS), Relieved by a Double-balloon Catheter, in a 68-year-old Woman

**DOI:** 10.5935/abc.20180151

**Published:** 2018-09

**Authors:** Edmar Atik, Alessandra Costa Barreto, Maria Angélica Binotto, Luiz Junya Kajita

**Affiliations:** Instituto do Coração do Hospital das Clínicas da Faculdade de Medicina, USP/, São Paulo, SP - Brazil

**Keywords:** Pulmonary Valve Stenosis, Cardiac Catheterization, Hypertrophy, Rigth Ventricular/physiopathology, Pulmonary Valve/surgery

## Clinical data

During a recent clinical evaluation for cholecystectomy surgery, a heart murmur was auscultated and complaint of fatigue at great efforts lasting for some months was reported by the patient. The diagnosis of severe PVS was attained, with a maximum gradient of 160 mmHg obtained at the echocardiography. The patient denied other symptoms and was unaware of the existence of this cardiopathy. She had no history of other morbidities and took vitamin D.

**Physical examination:** The patient was in good overall condition, eupneic, acyanotic, normal pulses in the 4 limbs. Weight: 70 kg, height: 160 cm, right upper limb blood pressure: 140/80 mmHg, heart rate: 80 bpm, oxygen saturation, 89%. Aorta not palpable in the suprasternal notch.

**Precordium:** Apex beat not palpable, discrete systolic impulses in the left sternal border (LSB). Muffled heart sounds, harsh systolic murmur, ++ / 4 in the pulmonary area, which irradiated to the entire LSB. The liver was not palpable, and the lungs were clear.

### Complementary exams

**Figure 1:**
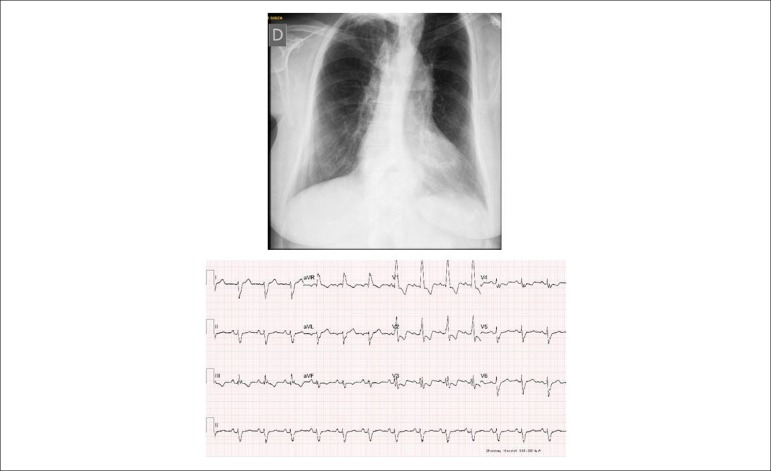
PA chest X-ray emphasizes normal cardiac area with prominent ventricular arch and slightly increased hilar pulmonary vascular network. Due to the marked increase of the pulmonary trunk, the middle arch draws attention due to the long concavity. The electrocardiogram highlights the signs of complete right bundle branch block.

**Electrocardiogram:** Sinus rhythm, 1^st^ degree atrioventricular block and complete right bundle branch block. PR: 0.22, QRS: 0.12, with rsR' complexes in V1 and RS in V6. T wave was negative from V1 to V4 and the S wave was prominent in the left precordial leads. AP = +80°, AQRS = +200°, AT = -20° (Figure 1).

**Chest radiography:** Normal cardiac area (cardiothoracic index of 0.50) with enhanced ventricular arch and excavated, rounded and long middle arch, with a more prominent hilar pulmonary vascular network (Figure 1).

**Echocardiogram:** Heart cavities with normal diameter, except for dilated right atrium. The right ventricle was hypertrophic, with systolic function preservation, with TAPSE = 1.8. Diastolic dysfunction was present, with relaxation alteration at tissue Doppler with Tei index = 0.62. The pulmonary valve was thickened with a dome-shaped opening and a maximum pressure gradient of 160 and a mean of 86 mmHg. Pulmonary regurgitation was mild. The pulmonary trunk was dilated, and the pulmonary arteries were confluent. A small 10-mm atrial septal defect allowed right-to-left shunting. Aorta = 28 mm, LA = 30, RV = 29, LV = 39, PT = 37, AP's = 20, septum = 8, posterior wall = 10 mm, LVEF = 72%, TV diameters = 31, MV = 20, PV = 20, Ao = 21 mm.

**Holter:** Sinus rhythm, frequent ventricular and atrial extrasystoles and episodes of nonsustained ventricular and supraventricular tachycardia.

**Clinical diagnosis:** Severe PVS with natural disease evolution, without heart failure and good physical tolerance.

**Clinical rationale:** In an adult patient with few symptoms, the recently observed clinical elements of systolic murmur in the pulmonary area, complete right bundle branch block and pulmonary trunk dilation, led to the diagnosis of PVS. This supposition was confirmed by the echocardiogram, with an adequate demonstration of the dome-shaped valve opening and marked right ventricular hypertrophy.

**Differential diagnosis:** Aortic valve stenosis is the most important differential diagnosis in this case, due to the location of the murmur and the patient's older age. However, the fact that the heart murmur did not radiate to the lateral neck sides rules out this diagnosis, as well as the occurrence of middle arch dilation on the chest radiography, and the right bundle branch block on the ECG.

**Conduct:** The immediate relief of the right ventricle overload was indicated, as it was shown to be of the utmost importance. Surgical intervention was ruled out due to the patient's age and the enthusiastic use of percutaneous procedures, proven effective even in adult patients.

Cardiac catheterization performed in the right heart confirmed the diagnosis of PVS of great impact. The cavitary pressures found were: RA = 20, RV = 160 / 8-22, PT = 30/20-23 mmHg. Systemic pressure was 110/60 mmHg. Marked right ventricular hypertrophy was observed, and the dome-shaped pulmonary valve opening was limited with a little reduced pulmonary annulus size.

**Figure 2:**
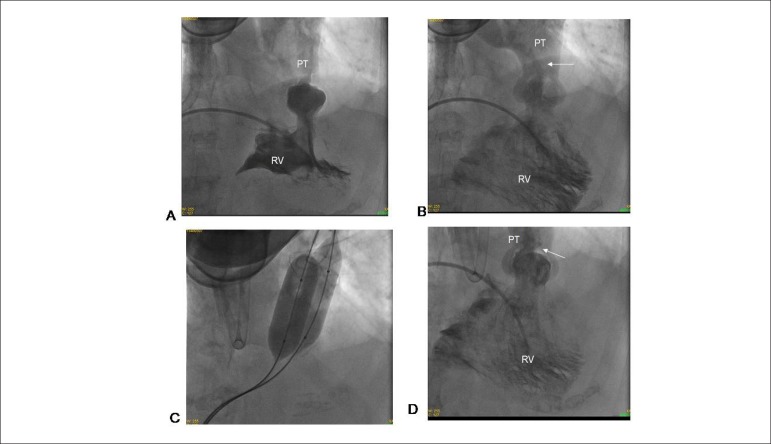
Cardiac angiography showing marked right ventricular hypertrophy in systole in A, and in diastole in B, with good ventricular contractility. Thinner contrast jet (arrow) passing through the stenotic pulmonary valve in B, before double-balloon valvuloplasty, in C, and after the procedure in D, with a thicker jet (arrow).

The pulmonary valvuloplasty was performed with two 20-and 18-mm balloons, which were inflated at the valve plane level, with the formation and disappearance of the hourglass image. Post-pulmonary valvuloplasty pressures were: RA = 12, RV = 80 /8-12, PT = 30 / 20-23 mmHg (Figure 2). Therefore, the procedure was considered very successful.

At the first reevaluation one week later, the patient showed clinical improvement, with more adequate breathing. The systolic murmur persisted in the pulmonary area, with lower intensity. The patient started receiving an adrenergic beta-blocker medication.

## Comments

Marked PVS has an unfavorable evolution, even at an early age in the neonatal period, as it can progress to sudden death due to right ventricular failure. After this age range, the disease evolution becomes more adequate, but right ventricular dysfunction, tricuspid valve regurgitation, arrhythmias and right heart failure appear in adulthood. These factors shorten patient survival to 30 to 40 years of age. Therefore, percutaneous pulmonary valvuloplasty has been indicated at early ages, aiming to prevent such unfavorable evolution. Thus, it can be affirmed that patient evolution in this clinical case is very peculiar, due to the marked degree of the congenital defect at a very old age. Also noteworthy is the rare occurrence of good ventricular function preservation and, as a result, the occurrence of few symptoms. A similar evolution has also been observed by other authors with percutaneous,^1,2^ as well as surgical treatment^3^ in adulthood. However, the literature shows that the effectiveness of percutaneous valvuloplasty becomes lower in adults (87%) in relation to younger ages (96%).^4^

## References

[1] Shrivastava S, Sundar AS, Rajani M (1989). Long-term efficacy of balloon pulmonary valvoplasty in late adult
life. Indian Heart J.

[2] Gibbs JL, Stanley CP, Dickinson DF (1986). Pulmonary balloon valvoplasty in late adult life. Int J Cardiol.

[3] Hirata N, Grimmig O, Minami K, Miche E, Körfer R (1997). Surgical repair of pulmonary stenosis with intact ventricular
septum in a 68-year-old woman. J Cardiovasc Surg (Torino).

[4] Holzer RJ, Gauvreau K, Kreutzer J, Trucco SM, Torres A, Shahanavaz S (2012). afety and efficacy of balloon pulmonary valvuloplasty: a
multicenter experience. Catheter Cardiovasc Interv.

